# Noncommunicable Disease Service Utilization among Expatriate Patients in Thailand: An Analysis of Hospital Service Data, 2014–2018

**DOI:** 10.3390/ijerph18189721

**Published:** 2021-09-15

**Authors:** Anon Khunakorncharatphong, Nareerut Pudpong, Rapeepong Suphanchaimat, Sataporn Julchoo, Mathudara Phaiyarom, Pigunkaew Sinam

**Affiliations:** 1 International Health Policy Program (IHPP), Ministry of Public Health, Nonthaburi 11000, Thailand; nareerut@ihpp.thaigov.net (N.P.); rapeepong@ihpp.thaigov.net (R.S.); sataporn@ihpp.thaigov.net (S.J.); mathudara@ihpp.thaigov.net (M.P.); pigunkaew@ihpp.thaigov.net (P.S.); 2 Educational Service Unit, Sirindhorn College of Public Health, Chonburi 20000, Thailand; 3 Division of Epidemiology, Department of Disease Control, Ministry of Public Health, Nonthaburi 11000, Thailand

**Keywords:** expatriates, utilization, healthcare access, health services, noncommunicable disease

## Abstract

Global morbidity associated with noncommunicable diseases (NCDs) has increased over the years. In Thailand, NCDs are among the most prevalent of all health problems, and affect both Thai citizens and non-Thai residents, such as expatriates. Key barriers to NCD health service utilization among expatriates include cultural and language differences. This study aimed to describe the situation and factors associated with NCD service utilizations among expatriate patients in Thailand. We employed a cross-sectional study design and used the service records of public hospitals from the Ministry of Public Health (MOPH) during the fiscal years 2014–2018. The focus of this study was on expatriates or those who had stayed in Thailand for at least three months. The results showed that, after 2014, there was an increasing trend in NCD service utilizations among expatriate patients for both outpatient (OP) and inpatient (IP) care. For OP care, Cambodia, Laos PDR, Myanmar, and Vietnam (CLMV) expatriates had fewer odds of NCD service utilization, relative to non-CLMV expatriates (*p*-value < 0.001). For IP care, males tended to have greater odds of NCD service utilization compared with females (AdjOR = 1.35, 95% CI = 1.05–1.74, *p*-value = 0.019). Increasing age showed a significant association with NCD service utilization. In addition, there was a growing trend of the NCD prevalence amongst expatriate patients. This issue points to a need for prompt public health actions if Thailand aims to have all people on its soil protected with universal health coverage for their well-being, as stipulated in the Sustainable Development Goals. Future studies that aim to collect primary evidence of expatriates at the household level should be conducted. Additional research on other societal factors that may help provide a better insight into access to healthcare for NCDs, such as socioeconomic status, beliefs, and attitudes, should be conducted.

## 1. Introduction

The World Health Organization (WHO) reports that noncommunicable diseases (NCDs) kill over 41 million people each year, equivalent to 71% of all deaths globally. Each year, 15 million people aged between 30 and 69 years die from NCDs and over 85% of these premature deaths occur in low- and middle-income countries [1]. Now the global movement on NCDs receives increasing support to tackle the problem from a number of parties. One of the most distinct examples of this is the United Nations, which set a target to reduce deaths from NCDs by 25% by 2025 [2]. The Sustainable Development Goals (SDGs) also have a target to achieve a decline of premature deaths from NCDs by one-third by 2030 [3].

NCDs are the leading health threat in Thailand. The prevalence of NCDs in Thailand has been increasing for several years, with about 320,000 deaths due to NCDs each year during 2014–2018, accounting for 75% of all Thai mortalities [4]. In 2018, the top three NCD-related causes of deaths were cancers, cerebrovascular diseases, and ischemic heart disease [5]. Therefore, many NCD prevention and control programs have been initiated. These include “Thailand Healthy Lifestyle Strategic Plan, B.E. 2011–2020” [6], “Proposal on Prevention and Control of NCDs during January 2017–December 2021” [7], and “Country Co-operation Strategy and Master Plan for Promoting National Physical Activity (2018–2030)” [8]. 

The public health problem relating to NCDs affects both the Thai population and non-Thai residents, such as expatriates. Currently, as of July 2021, according to the Foreign Workers Administration Office, the number of skilled workers were 139,748 (5.89%) of total migrant workers in Thailand [9]. Compared with the whole Thai population (66.8 million), this figure contributed to about 0.21% [10], However, it is important to note that this was the percentage of those possessing a work permit only. The data were not classified for the duration of holding a work permit or the duration of the stay in Thailand. Therefore, it is difficult to pinpoint the exact figure of those meeting the criteria of expatriates (a length of stay in the destination country of more than three months). Besides, there were many other types of expatriates whose data were not systematically collected by the public authorities, such as retired foreigners, overstayers, and oversea students. According to data from the Ministry of Labor (MOL) in 2021, there were 2.4 million expatriates who had obtained a work permit. Of these, most were migrant workers from Cambodia, Lao PDR, Myanmar, and Vietnam (so-called CLMV nations), accounting for 1.5 million (50% of the total) [9]. Cultural and language differences and unfamiliarity with the Thai healthcare systems serve as key barriers that hamper access to health services and other social supports for these people [11].

Thailand attempted to solve the problem of limited access to health services among CLMV migrants relatively well [12,13]. The Ministry of Public Health (MOPH) initiated the “Health Insurance Card Scheme (HICS)”, a public insurance arrangement for CLMV migrants in the informal sector in 2004. The HICS provides a comprehensive benefit package including outpatient care, inpatient care, emergency care, high-cost treatments, and health promotion [14]. However, when it comes to expatriates, who are not CLMV workers, it seems that the MOPH does not show clear direction. Note that, in principle, non-CLMV workers are still able to enroll in the Social Security Scheme (SSS)—a public insurance arrangement for workers in the formal sector, the same as a Thai citizen—or be insured by a private insurance scheme. These expatriates comprise a vast range of people, including professionals, students, and those marrying Thai citizens. Some literature refers to this group as “expatriates”, denoting a person leaving their place of birth to live in another country with the purpose of work, study, retirement, having a family, etc., and living in another country for more than three months [15].

NCDs are a major health problem among expatriates in other countries too. Studies in Qatar, Saudi Arabia, and the United Arab Emirates reported that NCDs were the most common health problem among expatriates (95.7%) [16,17,18]. Mishra et al. suggest that expatriates in the Middle East who had underlying NCDs might face difficulties in accessing health services as, in general, medical infrastructures were mainly prepared for domestic citizens [19]. 

Though there were some previous studies on low-skilled migrant workers from the neighboring countries of Thailand [20,21,22], the research on the health of expatriates is quite lacking. This research is probably amongst the first studies that explore service utilization of expatriates in public hospitals. The MOPH dataset comprises the data of public hospitals, which are the majority of all hospitals in Thailand (about 72.24% of the total). 

Therefore, the objectives of this study were to describe the situation of NCD service utilization and factors associated with utilization among expatriate patients in Thailand. It is hoped that the findings from this study can help enhance the academic value of research on the health of expatriates in Thailand and inform optimal policies to help Thailand achieve the SDGs, especially SDG3, ensure healthy lives, and promote wellbeing for all at all ages, so the country will leave no one behind regardless of citizenship status. 

## 2. Materials and Methods

### 2.1. Study Design and Data Source

We employed a cross-sectional study design, using secondary data. The database was the patient service records (both outpatient [23] and inpatient [IP] care) of the public hospitals affiliated to the MOPH, between 2014 and 2018. It should be noted that for IP records, data in 2016 were not available due to technical errors in providing the data to the MOPH. 

A few additional relevant points are as follows. First, all public hospitals are required to submit service data to the MOPH but for private hospitals, the submission is voluntary. To minimize selection bias, we limited the analysis to public hospitals only. Second, we excluded data from public hospitals in Bangkok. This is because public hospitals in Bangkok are not affiliated with the MOPH. Most of them are governed by the Bangkok Metropolitan Administration (but are still public) or are affiliated with the Ministry of Higher Education, Science, Research, and Innovation (university hospitals). For both types of hospitals, the requirement to submit patient data to the MOPH is voluntary, the same as private hospitals. Lastly, we excluded patients under 15 years of age from the analysis to avoid sparse data bias. 

### 2.2. Study Population

We focused on expatriate patients and examined NCD service utilization for Outpatient (OP) and inpatient (IP) care in public hospitals. The records of Thai patients and patients whose nationalities were not clearly identified were excluded. As we intended to focus on expatriate patients who had been residents in Thailand for lengthy periods, we then included only patients who had made at least two visits in a year, with the period between the first and the last visits being at least three months. This means that tourists and short-stay visitors were excluded. This followed the assumption that to be an expatriate, a length of stay in the receiving country for at least three months is required, as stipulated in previous literature [24]. In the end, there were data for a total of 145,726 OP patients and 3804 IP patients. The expatriate patients in the dataset were a mixture of expatriates from diverse nations and CLMV migrant workers. We then excluded people who we identified as CLMV workers (low-skilled migrant workers) but kept CLMV migrants or those who reported with the absence of a work permit in the analysis. Then, we used a nationality variable to classify the patients into CLMV and non-CLMV groups of expatriates.

Access to NCD services was quantified by the International Statistical Classification of Disease and Related Health Problems 10th Revision (ICD-10) [25]. The following ICD-10 codes were identified as NCD diagnoses: C00-D49; D50-D89; E00-E90; I00-I99; J00-J99, and K00-K93. We divided the hospital visit (outcome variable) into two groups: those with NCDs and those without. Demographic variables used in this study were gender, age group, nationality (CLMV versus non-CLMV), marital status, insurance status, service areas, and years of visit. We rearranged age variables into two groups: working-age adults (15–59 years), and the elderly (60 years and over). In addition, we stratified marital status into three levels: single, married, and others. Health insurance status was classified as insured (either by HICS or SSS or private insurance) and uninsured. The regional domicile was geographically divided into North, Central, Northeast, and South. 

### 2.3. Statistical Analyses

The analyses were divided into two parts: (i) descriptive statistics, and (ii) inferential analysis. For descriptive statistics, all categorical variables were shown in frequency and percentage, except age which was expressed in the form of median and percentile. The relationship between the demographic variables and NCD service utilization was determined by Chi-square test, Mann–Whitney U test, and multivariable logistic regression. Crude and adjusted odd ratios (OR) with 95% confidence interval (95% CI) were presented. The independent variables that exhibited a *p*-value of less than 0.2 in the bivariate analysis (for instance, by Chi-square test) were proceeded in the next step of analysis multivariable logistic regression. Before progressing to multivariable analysis, we assessed the effect of missing data by substituting methods and found that the complete-set analysis and the analysis on the dataset after replacing missing records did not show a marked difference. See Supplementary Materials for more details. All analyses were undertaken by STATA version 16.0 (serial number: 401406358220). The study was granted an ethics approval from the Institute for the Development of Human Research Protection, Thailand (letter head: IHRP 353/2563).

## 3. Results 

### 3.1. Outpatient Care

#### 3.1.1. Situation of Outpatient Service Utilizations among Expatriate Patients

**Figure 1:**
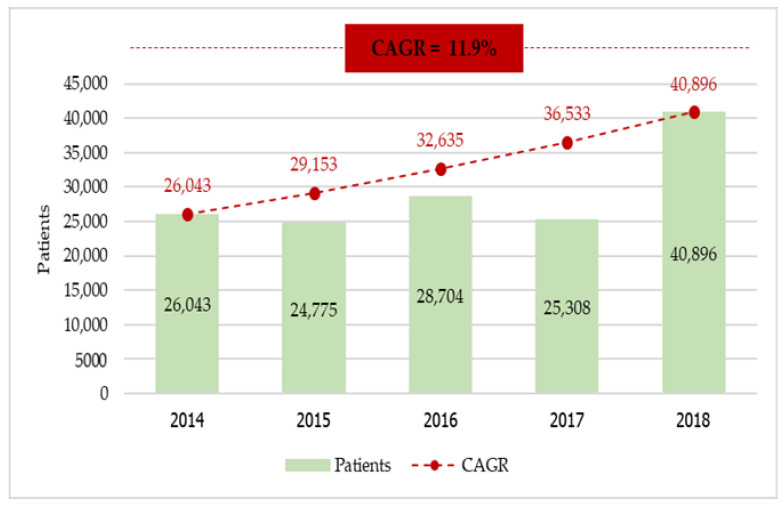
Number of expatriate outpatients in public hospitals in Thailand during 2014–2018.

In total, we acquired data for 145,726 expatriate patients from outpatient visits between the FY 2014 and 2018. As shown in Figure 1, the number of visits in public hospitals for OP care showed an increasing trend with a compound annual growth rate (CAGR) of 11.9%.

#### 3.1.2. Descriptive Statistics and Univariable Analysis

Table 1 revealed demographic characteristics of the patients receiving OP care between 2014 and 2018. Of 145,726 patients, 89,548 (61.4%) were involved with NCDs. The average age of patients with NCDs (34.5 years) was higher than those without (35.1 years). The elderly (60 years and over) saw a larger percentage of NCD diagnosis than the younger group (70.5%). About 50% of the patients attended OP services in the central region. Over 60% of the patients (either insured or uninsured) presented with NCDs. The number of patients enjoying OP services showed an increasing trend, ranging from 18.0 to 28.5% (for NCDs) and from 17.6 to 27.4% (for other diagnoses), see Table 1. 

#### 3.1.3. Multivariable Analysis

In the multivariable logistic regression for OP service, we found that being older showed a significant association with increasing NCD service access by about 1.34 times relative to the working age group (AdjOR = 1.34 95% CI: 1.28–1.41). Non-CLMV nationals were more likely to present with a NCD diagnosis at the point of care than CLMV nationals (*p*-value < 0.001). Married patients appeared to present with NCD diagnoses more frequently than single people. The service areas in the northern region tended to have higher access to NCD diagnosis than other regions (*p*-value < 0.001). The insured patients showed a more frequent NCD service access than the uninsured (*p*-value < 0.001). With reference to FY 2014, 2018 saw an increasing trend of NCD service utilizations (AdjOR = 1.04 95% CI: 1.01–1.08), see Table 2.

### 3.2. Inpatient Care

#### 3.2.1. Situation of Inpatient Service Utilization Expatriate Patients

**Figure 2:**
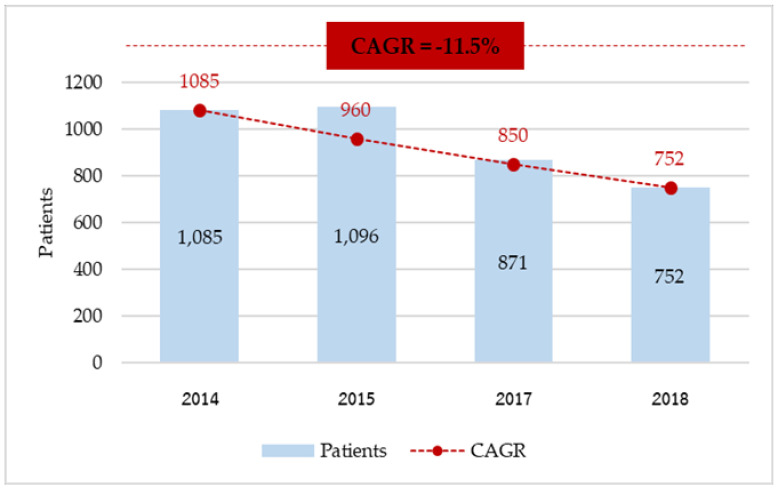
Number of expatriate inpatients in public hospitals in Thailand during 2014–2018.

In total, we acquired 3804 patients between FY 2014 and 2018. As shown in Figure 2, the number of patients attending public hospitals dropped from 1085 patients in 2014 to 752 patients in 2018, with the CAGR of −11.5% per year. 

#### 3.2.2. Descriptive Statistics and Univariate Analysis 

Table 3 presented demographic characteristics of expatriate inpatients between 2014 and 2018. Of 3804 records, 1837 encountered NCDs (48.3%). There was no difference between males and females regarding the presence of NCDs. The median age of expatriate patients with NCDs was 47 years, about 10 years older than other diagnoses. Non-CLMV nationals had a greater share for NCD diagnosis (56.8%), while the majority of CLMV nationals presented with non-NCDs. Only 39.0% of the patients in the southern region were diagnosed with NCDs, while for those in other regions, the percentage share varied between 42.8–60.1%. The relationship between NCD presentation and insurance status did not show a statistical significance; see Table 3. 

#### 3.2.3. Multivariable Analysis

The demographic variables used in the multivariable logistic regression analysis included gender, age group, nationality, service areas, and year of service. Females were found to have less frequent NCD service access compared with males (AdjOR = 0.49 95% CI: 0.42–0.57). The elderly appeared to have increasing numbers of NCD admissions compared to the working age group (AdjOR = 1.69 95% CI: 1.40–2.03). Likewise, increasing NCD care was presented in CLMV patients (AdjOR = 1.35 95% CI: 1.05–1.74), in comparison to other nationals. The patients in the northeast region exhibited a higher number of admissions than those in the north (AdjOR = 1.37 95% CI: 1.13–1.66). See Table 4.

## 4. Discussion 

Overall, this study found that in Thailand during 2014–2018, the access to NCD services among expatriate patients was relatively high, accounting for 61.4% of all OP care and 48.3% of IP admissions. Moreover, the trend of NCD service utilization continued to grow each year. NCD services in both OP and IP care were found to be more prevalent among females, the working age group, CLMV nationals, married patients, uninsured patients, and expatriates who received services in the central region. Factors that exhibited a positive correlation with NCD service utilization were gender, age group, nationality, and service areas for both OP and IP care. Marital status, insurance status, and year of visit exhibited a positive correlation with NCD service utilization for OP service only. 

These findings were consistent with several previous studies. For example, Nirwan and Singh found an increasing trend of NCD prevalence among Indian expatriates in Qatar [26]. Ahmed et al. reasoned that this was because expatriates were likely to be exposed to unhealthy food and therefore at risk of NCDs [27]. The increasing trends in NCD service utilization over time in both OP and IP services concurred with the report of Lee et al. that investigated the situation of NCD services in six countries (China, Ghana, India, Mexico, Russia and South Africa.) [28].

The finding that NCD service utilization was positively associated with the elderly age group was consistent with the study by Syed et al., which examined NCDs among expatriates in Qatar [16]. Similarly, a study performed in Beijing also found that expatriates aged above 55 years old (OR = 2.253) were more likely to seek NCD care [29]. This observation was explained by the nature of disease in that when people become older, they are more likely to be exposed to risk factors and develop a clinical syndrome of NCDs [30]. 

Our findings also suggested that females were more likely to have more frequent access to OP care for NCDs than males. This was in line with a previous study in Ethiopia by Abebe et al., which compared male expatriates with female expatriates and found that females were more likely to face NCD problems than males [31]. Evidence also showed that NCDs were the leading cause of deaths among women worldwide [32]. The possible explanation was that females were generally more vulnerable to risk factors of NCDS, such as sugary beverages, and physical inactivity compared to males [33]. However, Sriwanichakorn pointed out that higher access to NCD services among females was because they participated more in health examinations and screening than males. Males’ inattention to health care for NCDs resulted in worse clinical conditions of NCDs than women [34]. Thus, it is possible that whenever males access NCD services, they are prone to a more severe health status than females, and this usually requires IP care rather than OP care. The above reasons were consistent with our findings because we found that, for IP care, access to NCD services was more evident amongst males than females (but the inverse phenomenon was noticed in OP care).

Further, we found that the expatriates in the northern and northeastern regions mostly received NCD care to a greater extent than expatriates in other regions. A possible explanation was these regions of Thailand are a popular destination for long-stay expatriates. Some were retired men or businessmen who married Thai women, and therefore spent some time in Thailand every year [35]. Therefore, this may be the reason for a high level of expatriates access to NCD OP services [36,37]. Once married, most of these expatriates lived in the countryside, which was the home place of their Thai wives. Thus, when they were seriously ill, hospitalizations in public hospitals appeared to be more convenient than private hospitals, which were usually located in the city center [38]. 

Concerning nationality, CLMV nationals appeared have better access to NCD IP care. However, we observed a reverse finding in OP services where non-CLMV nationals had greater access to NCD care. This might be because CLMV nationals tend to live in congested conditions and suffer financial difficulties, meaning they avoid the use of NCD services if sickness is not serious [39]. In contrast to CLMV nationals, non-CLMV nationals (such as retired people from Europe or people from the Middle East) were more likely to pay for care whenever they faced minor illnesses. However, when illness was more serious and necessitated IP care, CLMV nationals tended to seek care at public hospitals, while non-CLMV nationals (who could afford the cost of care) tended to receive care at private hospitals [40].

With regard to health insurance status, the study found that more than half (60%) of expatriate patients did not possess any insurance. This finding was consistent with a study by Ratchanuch, et al., which explored the characteristics of expatriate patients who received medical services from Koh Phangan Hospital (one of the public hospitals in the touristic areas in the South of Thailand) during 2012–2014. The study found that uninsured expatriate workers accounted for 53.42% of all expatriate workers in Koh Phangan [41]. However, evidence from abroad found that less than half of expatriate workers were uninsured. A study among Korean expatriates in Vietnam, Cambodia, and Uzbekistan showed that only 26.6% had no health insurance [15]. Similarly, in Saudi Arabia, approximately 30% of expatriate employees were not yet enrolled in any health insurance scheme [42]. The fact that many expatriates in Thailand did not have any health insurance might be explained on many accounts. First, the campaign for public health insurance benefits provided in Thailand was quite limited and the enrolment in public insurance for expatriates (who were not CLMV workers) was voluntary. This in part created a perception of the low importance of public insurance enrolment among expatriates in the country [43]. In addition, even amongst CLMV workers, the prerequisite for possessing public health insurance was the acquisition of a work permit [44]. However, in reality, some expatriates were self-employed or did not enter Thailand in order to seek job prospects. Note that expatriates with better ability to pay can also opt to receive services from private hospitals. This implies that public hospitals become the main choice of care only for expatriates who have limited income, or who are uninsured.

Regarding methodological discussion, the major strength of this study was the use of actual individual service data from most of the public hospitals affiliated to the MOPH in Thailand. This helped present an overall picture of the situation of NCD service utilization among expatriate patients all over the country. However, some limitations remained. First, the use of in-service secondary data meant we could not control the quality of the data records. Although we have checked the effect of missing data and found that missing data had little effect on the main results, it is difficult to assess mis-recording, especially on the variables that involved the work status of expatriate patients. Second, some important variables, which were not a required input in the routine services, were absent. These included socioeconomic variables or household characteristics. Third, the approach of attempting to fulfill the criteria of expatriates (only those who had at least two visits over a one-year period, with an interval for the first and the last visit being at least 3 months) assured expatriate patients who were likely to have some time of stay in Thailand, but the trade-off was that we sacrificed a large number of patients and this definitely undermined the generalizability power of the analysis to a certain extent. Fourth, as this study used a cross-sectional approach, we could not make a strong causation inference from the findings. A population-based study that explores the service trend of a cohort of expatriates over time is recommended. Fifth, the lack of information about hospitals in Bangkok and private hospitals might cause a generalization problem and this limited a comparative analysis of utilization difference between urban settings such as Bangkok and rural communities. However, the findings presented also held some values. Even though the data used in this study did not consist of public hospitals in Bangkok and private hospitals in the country, the data represented the majority of public hospitals under MOPH and accounted for more than 70% of total hospitals in the country. The information used this study comprised 934 public hospitals affiliated with the MOPH, representing 72.24% of all hospitals in Thailand (1356 total hospitals) [45]. Hence we believed that this study’s results would reasonably reflect the situation of NCD service utilization among expatriate patients in Thailand under public services, though we recognized that there is room for improvement for future studies to include the data of private hospitals and also include many more hospitals in Bangkok.

Lastly, although we intended to exclude CLMV workers from the outset, we were not certain that those who were identified in the service records as workers were still holding a work permit (and at the same time, those identified as nonworkers might already possess a work permit at the time of receiving care). This is because the MOPH dataset was not automatically linked with the MOL dataset. Hence, the identification of the working status of a patient was performed at the point of care by self-reporting by the patient or through a verbal interview conducted by the healthcare provider.

We recommend that future studies should include information from private hospitals in order to better elaborate on the situation of OP and IP visits among expatriates over the whole country. These kinds of studies can also enable us to collate and compare the service difference between public and private hospitals. In addition, this study only described the utilization in health facilities. Future primary studies that explore the need for services at a household level would be extremely useful as many more unobserved factors (such as socioeconomic status, unmet need, beliefs, and attitudes) can be collected.

## 5. Conclusions

This study reaffirms that NCDs are a growing public health concern amongst expatriates in Thailand. The share of NCD diagnosis among expatriate patients was relatively high, equivalent to 61.4% of all OP care and 48.3% of IP admissions. Factors demonstrating a positive relationship with NCD utilization for both OP and IP care were gender, age group, nationality, and service areas. Marital status, insurance status, and years of visit showed a positive correlation with NCD utilization for OP service only. Thus, policies and public health interventions that aim to mitigate NCD problems and facilitate access to NCD care among expatriate patients in Thailand should be promptly introduced.

## Figures and Tables

**Table 1:** Demographic characteristics of expatriate patients attending OP services in Thailand, 2014–2018.

Characteristics	With NCDs N = 89,548	Without NCDs N = 56,178	Test (*p*-Value)
Gender—*n* (%)			Chi-square < 0.001
Male	37,105 (59.1)	25,696 (40.9)	
Female	52,442 (63.2)	30,482 (36.8)	
Median age—years (P25, P75)	34.5 (26.9,45.9)	35.1 (25.4,41.3)	Mann–Whitney U < 0.001
Age group—*n* (%)			Chi-square < 0.001
15–59 years	80,744 (60.6)	52,495 (39.4)	
60 years and over	8776 (70.5)	3665 (29.5)	
Nationality—*n* (%)			Chi-square < 0.001
CLMV	82,525 (61.0)	52,867 (39.0)	
Non-CLMV	7023 (68.0)	3311 (32.0)	
Marital status—*n* (%)			Chi-square < 0.001
Single	35,021 (58.7)	24,615 (41.3)	
Married	49,160 (63.0)	28,873 (37.0)	
Other	1446 (73.7)	517 (26.3)	
Service areas—*n* (%)			Chi-square < 0.001
North	19,588 (66.0)	10,098 (34.0)	
Central	43,886 (59.6)	29,783 (40.4)	
Northeast	11,550 (64.4)	6393 (35.6)	
South	14,524 (59.5)	9904 (40.5)	
Insurance status—*n* (%)			Chi-square < 0.001
Insured	31,045 (62.1)	18,922 (37.9)	
Uninsured	58,003 (61.1)	36,948 (38.9)	
Year of service—*n* (%)			Chi-square < 0.001
2014	16,135 (62.0)	9908 (38.0)	
2015	15,109 (61.0)	9666 (39.0)	
2016	17,310 (60.3)	11,394 (39.7)	
2017	15,515 (61.3)	9793 (38.7)	
2018	25,479 (62.3)	15,417 (37.7)	

**Table 2:** Factors associated with NCD OP visits among expatriate patients in Thailand, 2014–2018.

Factors	Bivariate Analysis by Chi Square Test		Multivariable Logistic Regression	
	Crude OR (95% CI)	*p*-Value	Adjusted OR (95% CI)	*p*-Value
Female (vs. Male)	1.19 (1.16–1.21)	<0.001	1.17 (1.14–1.20)	<0.001
60 years and over (vs. 15–59 years)	1.56 (1.50–1.62)	<0.001	1.34 (1.28–1.41)	<0.001
CLMV (vs. non-CLMV)	0.74 (0.71–0.77)	<0.001	0.84 (0.80–0.88)	<0.001
Married status (vs. Single)				<0.001
Married	1.20 (1.17–1.22)	<0.001	1.08 (1.05–1.10)	<0.001
Other	1.97 (1.78–2.18)	<0.001	1.51 (1.36–1.67)	<0.001
Service areas (vs. North)				<0.001
Central	0.76 (0.74–0.78)	<0.001	0.81 (0.79–0.84)	<0.001
Northeast	0.93 (0.90–0.97)	0.001	0.92 (0.88–0.96)	<0.001
South	0.76 (0.73–0.78)	<0.001	0.82 (0.79–0.85)	<0.001
Uninsured (vs. insured)	0.96 (0.94–0.98)	<0.001	0.93 (0.90–0.95)	<0.001
Year of service (vs. 2014)				<0.001
2015	0.96 (0.93–0.99)	0.023	0.98 (0.95–1.02)	0.319
2016	0.93 (0.90–0.97)	<0.001	0.96 (0.92–0.99)	0.013
2017	0.97 (0.94–1.01)	<0.295	1.00 (0.96–1.03)	0.842
2018	1.01 (0.98–1.05)	<0.460	1.04 (1.01–1.08)	0.013

**Table 3:** Demographic characteristics of expatriate inpatients during 2014–2018.

Characteristics	With NCDs N = 1837	Without NCDs N = 1967	Test (*p*-Value)
Gender—*n* (%)			Chi-square < 0.001
Male	865 (59.6)	586 (40.4)	
Female	972 (41.3)	1381 (58.7)	
Median age—years (P25, P75)	47 (32.4, 60.2)	37.9 (25.2, 46.6)	Mann–Whitney U < 0.001
Age group—*n* (%)			Chi-square < 0.001
15–59 years	1357 (44.6)	1687 (55.4)	
60 years and over	480 (63.2)	279 (36.8)	
Nationality—*n* (%)			Chi-square 0.001
CLMV	1633 (47.4)	1812 (52.6)	
Non-CLMV	204 (56.8)	155 (43.2)	
Marital status—*n* (%)			Chi-square < 0.295
Single	541 (49.0)	562 (51.0)	
Married	1248 (47.9)	1360 (52.1)	
Other	27 (58.7)	19 (41.3)	
Service areas—*n* (%)			Chi-square < 0.001
North	443 (51.9)	410 (48.1)	
Central	566 (42.8)	755 (57.2)	
Northeast	548 (60.1)	364 (39.9)	
South	280 (39.0)	438 (61.0)	
Insurance status—*n* (%)			Chi-square < 0.418
Insured	501 (47.3)	558 (52.7)	
Uninsured	1334 (48.8)	1401 (51.2)	
Year of service—*n* (%)			Chi-square < 0.144
2014	515 (47.5)	570 (52.5)	
2015	509 (46.4)	587 (53.6)	
2017	389 (51.7)	363 (48.3)	
2018	515 (47.5)	570 (52.5)	

**Table 4:** Factors associated with NCD IP care among expatriate patients in Thailand, 2014–2018.

Factors	Bivariate Analysis by Chi Square Test		Multivariable Logistic Regression	
	Crude OR (95% CI)	*p*-Value	Adjusted OR (95% CI)	*p*-Value
Female (vs. Male)	0.48 (0.42–0.55)	<0.001	0.49 (0.42–0.57)	<0.001
60 years and over (vs. 15–59 years)	2.14 (1.82–2.52)	<0.001	1.69 (1.40–2.03)	<0.001
CLMV (vs. non-CLMV)	0.69 (0.55–0.85)	<0.001	1.35 (1.05–1.74)	0.019
Service areas (vs. North)				<0.001
Central	0.69 (0.58–0.82)	<0.001	0.73 (0.61–0.87)	<0.001
Northeast	1.40 (1.16–1.69)	<0.001	1.37 (1.13–1.66)	0.002
South	0.59 (0.48–0.72)	<0.001	0.63 (0.52–0.78)	<0.001
Year of service (vs. 2014)				0.177
2015	0.95 (0.81–1.13)	0.618	1.04 (0.87–1.24)	0.675
2017	1.05 (0.88–1.25)	0.607	1.11 (0.92–1.34)	0.268
2018	1.18 (0.98–1.43)	0.075	1.23 (1.01–1.49)	0.037

## Data Availability

Data available on request due to ethical restrictions.

## Supplementary Materials

The following are available online at https://www.mdpi.com/article/10.3390/ijerph18189721/s1, Table S1: Factors associated with NCD service utilization for OP visits among expatriate patients in Table 2014–2018; Table S2: Factors associated with NCD service utilization in IP care among expatriate patients in Table 2014–2018; Table S3: Factors associated with NCD service utilization for OP visits among expatriate patients in Thailand, 2014–2018; Table S4: Factors associated with NCD service utilization in IP care among expatriate patients in Thailand, 2014–2018.
