# Supplementary material for: Noncommunicable Disease Service Utilization among Expatriate Patients in Thailand: An Analysis of Hospital Service Data, 2014–2018
Source: Int J Environ Res Public Health. 2021 Sep 15;18(18):9721. doi: 10.3390/ijerph18189721 (PMC8472549; doi:10.3390/ijerph18189721)
Supplement: Supplementary file 1 [file ijerph-18-09721-s001.zip › ijerph-1319063-supplementary.pdf]

**Table S1.** Factors associated with NCD service utilization for OP visits among expatriate patients in Table 2014-2018. (Substituting missing data with the value that showed percentage majority).

| Factors | Bivariate analysis by Chi square test | | Multivariable logistic regression | |
|---|---|---|---|---|
| | Crude OR (95% CI) | *P*-value | Adjusted OR (95% CI) | *P*-value |
| Female (vs. male) | 1.19 (1.16–1.22) | <0.001 | 1.17 (1.15–1.20) | <0.001 |
| Age group (vs. 15 – 59 years) | | | | <0.001 |
| 60 years and over | 1.56 (1.50–1.62) | <0.001 | 1.35 (1.29–1.41) | <0.001 |
| CLMV (vs. non-CLMV) | 0.74 (0.71–0.77) | <0.001 | 0.84 (0.8–0.88) | <0.001 |
| Married status (vs. Single) | | | | <0.001 |
| Married | 1.20 (1.18–1.23) | <0.001 | 1.09 (1.06–1.11) | <0.001 |
| Other | 1.97 (1.78–2.18) | <0.001 | 1.52 (1.37–1.69) | <0.001 |
| Service areas (vs. North) | | | | <0.001 |
| Central | 0.76 (0.74–0.78) | <0.001 | 0.82 (0.79–0.84) | <0.001 |
| North Eastern | 0.93 (0.90–0.97) | 0.001 | 0.92 (0.88–0.95) | <0.001 |
| South | 0.76 (0.73–0.78) | <0.001 | 0.82 (0.79–0.85) | <0.001 |
| Uninsured (vs. insured) | 0.96 (0.94–0.98) | <0.001 | 0.93 (0.91–0.95) | <0.001 |
| Year of service (vs. 2014) | | | | <0.001 |
| 2015 | 0.96 (0.93–0.99) | 0.025 | 0.99 (0.95–1.02) | 0.420 |
| 2016 | 0.93 (0.90–0.97) | <0.001 | 0.96 (0.93–0.99) | 0.016 |
| 2017 | 0.97 (0.94–1.01) | 0.130 | 0.99 (0.96–1.03) | 0.745 |
| 2018 | 1.01 (0.98–1.05) | 0.367 | 1.05 (1.02–1.08) | 0.003 |

**Table S2.** Factors associated with NCD service utilization in IP care among expatriate patients in Table 2014-2018. (Substituting missing data with the value that showed percentage majority).

| Factors | Bivariate analysis by Chi square test | | Multivariable Logistic regression | |
|---|---|---|---|---|
| | Crude OR (95% CI) | *P*-value | Adjusted OR (95% CI) | *P*-value |
| Female (vs. male) | 0.48 (0.42–0.55) | <0.001 | 0.49 (0.43–0.57) | <0.001 |
| 60 years and over (vs. 15 – 59 years) | 2.14 (1.82–2.52) | <0.001 | 1.69 (1.41–2.04) | <0.001 |
| CLMV (vs. non-CLMV) | 0.68 (0.55–0.85) | 0.001 | 1.35 (1.05–1.74) | 0.019 |
| Service areas (vs. North) | | | | <0.001 |
| Central | 0.69 (0.58–0.82) | <0.001 | 0.73 (0.61–0.87) | <0.001 |
| North Eastern | 1.39 (1.15–1.68) | 0.001 | 1.37 (1.12–1.66) | 0.002 |
| South | 0.59 (0.48–0.72) | <0.001 | 0.63 (0.52–0.78) | <0.001 |
| Year of service (vs. 2014) | | | | 0.172 |
| 2015 | 0.96 (0.81–1.14) | 0.632 | 1.04 (0.87–1.24) | 0.660 |
| 2017 | 1.05 (0.88–1.25) | 0.593 | 1.11 (0.92–1.34) | 0.260 |
| 2018 | 1.19 (0.98–1.43) | 0.072 | 1.23 (1.01–1.49) | 0.036 |

**Table S3.** Factors associated with NCD service utilization for OP visits among expatriate patients in Thailand, 2014–2018 (Substituting missing data with the value that showed percentage minority).

| Factors | Bivariate analysis by Chi square test | | Multivariable logistic regression | |
|---|---|---|---|---|
| | Crude OR (95% CI) | *P*-value | Adjusted OR (95% CI) | *P*-value |
| Female (vs. male) | 1.19 (1.17–1.22) | <0.001 | 1.17 (1.15–1.20) | <0.001 |
| Age group (vs. 15 – 59 years) | | | | <0.001 |
| 60 years and over | 1.55 (1.49–1.62) | <0.001 | 1.37 (1.31–1.43) | <0.001 |
| CLMV (vs. non-CLMV) | 0.74 (0.71–0.77) | <0.001 | 0.84 (0.8–0.88) | <0.001 |
| Married status (vs. Single) | | | | <0.001 |
| Married | 1.20 (1.17–1.22) | <0.001 | 1.08 (1.05–1.1) | <0.001 |
| Other | 1.40 (1.34–1.47) | <0.001 | 1.24 (1.18–1.3) | <0.001 |
| Service areas (vs. North) | | | | <0.001 |
| Central | 0.76 (0.74–0.78) | <0.001 | 0.82 (0.79–0.84) | <0.001 |
| North Eastern | 0.93 (0.90–0.97) | 0.001 | 0.91 (0.88–0.95) | <0.001 |
| South | 0.76 (0.73–0.78) | <0.001 | 0.81 (0.78–0.84) | <0.001 |
| Uninsured (vs. insured) | 0.96 (0.94–0.98) | <0.001 | 0.93 (0.91–0.95) | <0.001 |
| Year of service (vs. 2014) | | | | <0.001 |

| | | | | |
|---|---|---|---|---|
| 2015 | 0.96 (0.93–0.99) | 0.025 | 0.98 (0.95–1.02) | 0.406 |
| 2016 | 0.93 (0.90–0.97) | <0.001 | 0.96 (0.93–0.99) | 0.020 |
| 2017 | 0.97 (0.94–1.01) | 0.130 | 0.99 (0.96–1.03) | 0.778 |
| 2018 | 1.01 (0.98–1.05) | 0.367 | 1.05 (1.02–1.08) | 0.003 |

**Table S4.** Factors associated with NCD service utilization in IP care among expatriate patients in Thailand, 2014–2018 (Substituting missing data with the value that showed percentage minority).

| **Factors** | **Bivariate analysis by Chi square test** | | **Multivariable Logistic regression** | |
|---|---|---|---|---|
| | **Crude OR (95% CI)** | ***P*-value** | **Adjusted OR (95% CI)** | ***P*-value** |
| Female (vs. male) | 0.48 (0.42–0.54) | <0.001 | 0.49 (0.43–0.57) | <0.001 |
| 60 years and over (vs. 15 – 59 years) | 2.14 (1.82–2.52) | <0.001 | 1.68 (1.40–2.03) | <0.001 |
| CLMV (vs. non-CLMV) | 0.68 (0.55–0.85) | 0.001 | 1.35 (1.05–1.74) | 0.021 |
| Service areas (vs. North) | | | | <0.001 |
| Central | 0.69 (0.58–0.82) | <0.001 | 0.73 (0.61–0.87) | <0.001 |
| North Eastern | 1.39 (1.15–1.68) | 0.001 | 1.37 (1.12–1.66) | 0.002 |
| South | 0.59 (0.48–0.72) | <0.001 | 0.63 (0.52–0.78) | <0.001 |
| Year of service (vs. 2014) | | | | 0.171 |
| 2015 | 0.96 (0.81–1.14) | 0.632 | 1.04 (0.87–1.24) | 0.657 |
| 2017 | 1.05 (0.88–1.25) | 0.593 | 1.11 (0.92–1.34) | 0.259 |
| 2018 | 1.19 (0.98–1.43) | 0.072 | 1.23 (1.01–1.50) | 0.035 |
